# Cell-Free Circulating Methylated SEPT9 for Noninvasive Diagnosis and Monitoring of Colorectal Cancer

**DOI:** 10.1155/2018/6437104

**Published:** 2018-04-23

**Authors:** Bo Fu, Peng Yan, Shan Zhang, Yan Lu, Li Pan, Wenqiang Tang, Shen Chen, Shuangfeng Chen, Anqi Zhang, Wei Liu

**Affiliations:** ^1^Department of Central Laboratory, Liaocheng People's Hospital, Liaocheng, China; ^2^Department of Gastroenterology, Liaocheng People's Hospital, Liaocheng, China; ^3^Department of Breast and Thyroid Surgery, Liaocheng People's Hospital, Liaocheng, China

## Abstract

Identification of early-stage tumor and monitoring therapeutic efficacy and recurrence or metastasis of colorectal cancer (CRC) are urgently warranted for improving the outcome of CRC patients and reducing the disease-related mortality. In this study, we evaluated the diagnostic value of cell-free circulating methylated SEPT9 (mSEPT9) for CRC and beyond CRC and examined the potentiality of mSEPT9 in assessing therapeutic efficacy and monitoring recurrence of CRC. Our results confirmed the favorable diagnostic value of plasma mSEPT9 for CRC, with a sensitivity of 61.22% (95% confidence interval (CI): 51.33%–70.27%) and specificity of 93.7% (95% CI: 91.09%–95.57%) using 2/3 algorithm. The positive rate of mSEPT9 in CRC was correlated with tumor size, histological grade, and general histological type (*P* < 0.05). Beyond CRC, gastric cancer patients also presented a high positive rate of plasma mSEPT9 (70%). The conversions between preoperative and postoperative plasma mSEPT9 reflected the therapeutic efficacy of curatively intended surgery for CRC patients. The persistent positivity of plasma mSEPT9 after surgery (within 7–14 days) was highly associated with impending recurrences or metastases (within one year), with a sensitivity of 100%. Postoperative mSEPT9 status during follow-up also provided valuable indication for CRC recurrence or metastases, with a good consistency (kappa = 0.818, *P* = 0.001). Our results verified the reliability of plasma mSEPT9 as a biomarker for noninvasive diagnosis of CRC. More significantly, we revealed its valuable role in appraising CRC therapeutic efficacy and monitoring CRC recurrences or metastases. Further studies with larger sample sizes are needed to verify and elucidate the clinical utility of the promising findings.

## 1. Introduction

Colorectal cancer (CRC) is the third most frequently diagnosed malignancy, accounting for approximately 10% of global cancer burden [1–3]. Survival of CRC patients is significantly associated with the staging of the disease at diagnosis. The five-year survival rate for CRC patients diagnosed in early stage is >90%, while for those diagnosed in late stage is approximately 7% [4]. Therefore, early diagnosis and management of CRC are pivotal in improving treatment outcomes for CRC patients and reducing the disease-related mortality. Currently, there are several approaches to screen CRC, such as fecal occult blood test (FOBT) and colonoscopy. FOBT is a noninvasive and low-cost method, but its sensitivity for CRC is limited [5]. Colonoscopy is the gold standard for CRC screen with a specificity of >95%, but it requires bowel preparation and occasionally accompanied with severe complications [6]. Since the conventional methods for CRC screening are either ineffective or invasive, more patient-friendly and less-invasive approaches with high sensitivity and specificity are imperative.

Most of early-stage CRC patients undergo curatively intended surgery to remove primary lesions and regional lymph nodes metastases [7]. However, despite the thoroughness of initial radical resection, 30%–50% patients would confront CRC recurrences and die of metastases [8]. Detection of CRC recurrences or metastases in early stage may improve long-term outcomes through timely treatment. Periodic computed tomography (CT) scan and serum carcinoembryonic antigen (CEA) measurement are the most common methods to monitor CRC recurrences [9]. However, CT scans have limited sensitivity for small lesions (<1 cm in diameter) and high false positive rate [3, 10]. CEA test is currently the only blood-based methods recommended for routinely monitoring CRC recurrences, but its sensitivity and specificity are suboptimal [11]. Development of novel sensitive biomarkers for assessing occult residual diseases after surgery and monitoring recurrences or metastases of CRC are therefore urgently needed.

Tumor-specifically altered DNA releases into plasma and constitutes circulating tumor DNA (ctDNA), which could be utilized for noninvasive detection and monitoring of tumor burden [12–14]. Given that mutations occur only in 5 to 50% cancer cells, mutational hotspots are not easy to be identified in plasma. While hypermethylation in promoter region of multiple tumor genes could be stably detected in most cancers (>90%), which makes it a feasible and specific biomarker [15]. Mounting evidence indicate that altered DNA methylation is one of the most common aberrant epigenetic modifications, which play essential roles in CRC initiation and progression [16, 17]. Therefore, CRC specifically methylated cell-free DNA in plasma may serve as a putative biomarker for early detection, therapeutic efficacy assessment, and recurrence monitoring.

*SEPT9* gene locates at chromosome 17q25.3 and encodes Septin9 protein, which is a GTP-binding protein, and plays crucial physiological roles in actin dynamics, microtubule regulation, cytoskeletal remodeling, vesicle trafficking, and exocytosis [18, 19]. *SEPT9* gene has 18 distinct transcripts generated by alternative splicing and encodes 15 polypeptides [20]. Hypermethylation of the v2 transcript of *SEPT9* gene occurs only in colorectal adenomas and cancer, while the other *SEPT9* transcripts were either not methylated or methylated in both cancer and normal cells [21]. Aberrant methylation of v2 transcript has been observed in almost 100% CRC tissues, leading to significantly decreased *SEPT9* expression in colon neoplastic progression [22]. Emerging results have shown that cell-free circulating methylated SEPT9 (mSEPT9) is a promising biomarker for CRC detection [23–25], and Epi proColon 2.0 kit for mSEPT9 detection has been developed and approved by the US Food and Drug Administration (FDA). However, the correlation between clinicopathologic characteristics and plasma mSEPT9 in CRC has been rarely reported, and it is also unclear that whether mSEPT9 could be used as an indicator for monitoring tumor burden. In this study, we evaluated the diagnostic value of plasma mSEPT9 for CRC and beyond CRC and then investigated the associations between clinicopathologic characteristics and mSEPT9. Additionally, we examined the feasibility of mSEPT9 in CRC therapeutic efficacy assessment and recurrence monitoring.

## 2. Materials and Methods

### 2.1. Ethics, Consent, and Permissions

The study was approved by the ethics committee of Liaocheng People's Hospital. All procedures performed in studies involving human participants were in accordance with the ethical standards of the institutional and/or national research committee and with the 1964 Helsinki declaration and its later amendments or comparable ethical standards. Informed consents were obtained from all individual participants included in the study.

### 2.2. Patients and Samples

To evaluate the diagnostic value of plasma mSEPT9, a total of 558 subjects were enrolled in this study, including 98 samples with CRC, 101 samples with adenoma, 76 samples with noncolorectal cancers, 30 samples with inflammation, and 253 subjects with no evidence of diseases (NED) (Table 1). The plasma specimens were collected before intervention at Liaocheng People's Hospital from July 2015 to December 2016. All cancer cases were confirmed by histopathologic examination. TNM staging of CRC patients was determined according to the 7th edition of the American Joint Committee on Cancer (AJCC) cancer staging manual [26]. The healthy control specimens were collected from healthy individuals without neoplasms.

To evaluate the potential of mSEPT9 for monitoring therapeutic efficacy of CRC, a total of 19 cases treated with curatively intended surgery were enrolled. The plasma specimens were collected before surgery and within 7–14 days after surgery without chemotherapy or radiotherapy at Liaocheng People's Hospital between July 2015 and June 2016. CRC impending recurrences or metastases were recorded during one-year postoperative follow-up.

To evaluate the potential of mSEPT9 for monitoring recurrences or metastases of CRC, we enrolled 16 patients, who were either recently diagnosed and underwent initial treatment or had been monitored for CRC recurrence. Follow-up information, including the date of surgery, adjuvant treatment strategy, and recurrence status, were collected. Recurrences or metastases were determined based on diagnostic tests (CT scan, magnetic resonance imaging (MRI), positron emission tomography (PET) scan, or colonoscopy) and confirmed by tissue pathology when available [11].

### 2.3. Plasma Preparation and Storage

Peripheral blood samples were collected in 10 mL K_2_EDTA tubes (Vacutainer, Becton Dickinson, New Jersey, USA) and kept at 4°C (<4 h) before plasma processing. Plasma was obtained by repeated centrifugation at 1250 rcf for 12 min at 4°C (at the lowest deceleration) and stored at −80°C until used.

### 2.4. Cell-Free DNA Extraction and Bisulfite Conversion

Plasma cell-free DNA extraction and bisulfite conversion were performed using Epi proColon 2.0 kit (Epigenomics AG, Berlin, Germany), according to the manufacturer's instructions. Briefly, 3.5 mL plasma was mixed with equal volume of lysis buffer and incubated for 10 min. Subsequently, magnetic beads and absolute ethanol were added and thoroughly rotated for 45 min. After washing, the purified DNA was eluted from the magnetic beads and treated with bisulfite reagents at 80°C for 45 min. The bisulfite modified DNA (bisDNA) was captured by magnetic beads and eluted in 60 *μ*L buffer after washing.

### 2.5. Methylated SEPT9 Detection

Methylated SEPT9 was detected by real-time PCR using the PCR reagents in Epi proColon 2.0 kit (Epigenomics AG, Berlin, Germany), according to the manufacturer's instructions. Briefly, PCR was performed in triplicate with 15 *μ*L DNA per reaction. The sequences of primers, blockers, and probes for mSEPT9 detection were described previously [27]. The PCR program was set as follows: activation at 94°C for 20 min; 50 cycles at 62°C for 5 s, 55.5°C for 35 s, and 93°C for 30 s; and cooling at 40°C for 5 s. ACTB (*β*-actin) served as an internal reference to assess the integrity of each reaction. Amplification curves for each reaction were manually verified by two independent reviewers. Positivity of each reaction was determined according to the manufacturer's instructions, and the results of each sample were analyzed using either 2/3 algorithm or 1/3 algorithm, as described previously [6].

### 2.6. CEA Assay

The concentration of serum CEA was measured using the electrochemiluminescence immunoassay (Roche, Mannheim, Germany), as described previously [28]. CEA level of 5 ng/mL or above was defined as positive, according to the manufacturer's instructions.

### 2.7. Statistical Analysis

All statistical analyses were performed using SPSS18 software (SPSS Inc., Chicago, USA). Frequency distributions were compared using chi-square or Fisher exact test (when appropriate). Kappa test was used to assess agreement between mSEPT9 status and CRC recurrence or metastasis. Sensitivity was measured as a proportion of true positive cases to the number of CRC cases. Specificity was measured as a proportion of true negative cases to the number of controls. Positive predictive value (PPV) was measured as a proportion of true positive cases to the number of cases with positive mSEPT9. Negative predictive value (NPV) was measured as a proportion of true negative cases to the number of cases with negative mSEPT9. Binomial distribution was assumed for calculations of 95% confidence interval (CI). Receiver operating characteristic (ROC) curves were calculated based on the cycle threshold (Ct) values of mSEPT9 to evaluate its feasibility in differentiating the subgroups. Ct values were set as 50 (the maximal PCR cycle number) for the undetermined samples as described previously [27]. Area under the ROC curve (AUC) was calculated. *P* < 0.05 was considered statistically significant.

## 3. Results

### 3.1. Diagnostic Value of Plasma mSEPT9 for CRC and beyond CRC

**Figure 1:**
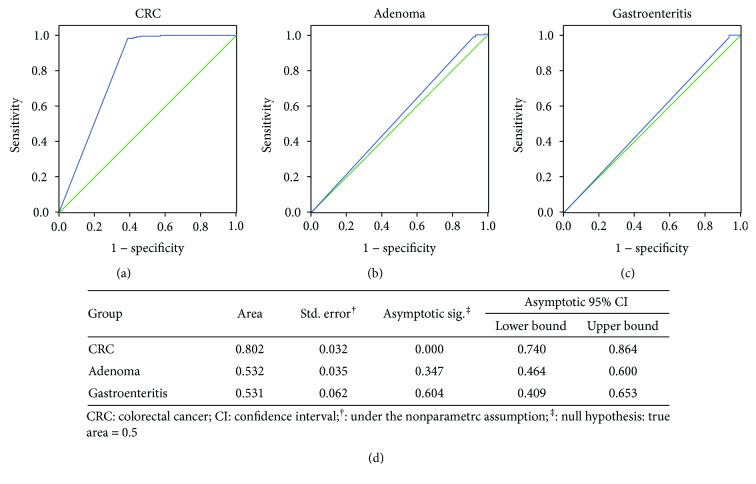
ROC curves of plasma mSEPT9 for predicting (a) colorectal cancer (CRC), (b) adenoma, and (c) gastroenteritis. The curves were generated using mean threshold count (Ct) values of mSEPT9 in each disease versus that in patients with no evidence of disease (NED) using 2/3 algorithm. (d) Area under the curve (AUC) of the mSEPT9 test in (a) CRC, (b) adenoma, and (c) gastroenteritis.

To evaluate the diagnostic value of circulating mSEPT9 for CRC and beyond CRC, we collected 558 plasma specimens, including CRC (*n* = 98), adenoma (*n* = 101), noncolorectal cancers (*n* = 76), inflammation (*n* = 30), and healthy controls (NED) (*n* = 253). The demographic and clinicopathologic characteristics of these enrolled subjects were presented in Table 1. Plasma mSEPT9 status of these samples were detected using real-time PCR in triplicate and defined as positive at 2/3 algorithm (a high-specificity approach). The results showed that mSEPT9 was positive in 61.2% (60/98) of CRC cases while only 1.6% in NED (*P* < 0.001; Table 2). The ROC curve was plotted in Figure 1(a), and the calculated AUC was 0.802 (95% CI: 0.740–0.864) (Figure 1(d)), suggesting significantly distinguished mSEPT9 status in CRC from NED. The sensitivity, specificity, PPV, and NPV of mSEPT9 for CRC using 2/3 algorithm were 61.22% (95% CI: 51.33%–70.27%), 98.42% (95% CI: 96.01%–99.38%), 93.75% (95% CI: 85%–97.54%), and 86.76% (95% CI: 82.35%–90.2%), respectively (Table 3).

Given that CRC usually evolves from adenoma [29], we sought to examine mSEPT9 status in patients with precancerous lesions. Adenoma cases showed a mSEPT9 positivity of 7.9% (Table 2), which was significantly lower than that of CRC (61.2%) (*P* < 0.001; Table 2). ROC curve showed a suboptimal performance of mSEPT9 for adenoma diagnosis, with an AUC of 0.532 (95% CI: 0.464–0.600) (Figures 1(b) and 1(d)), suggesting its inadaptability for adenoma diagnosis.

There are limited publications reporting the diagnostic value of plasma mSEPT9 in other diseases beyond CRC; therefore, we conducted mSEPT9 detection in patients with noncolorectal cancers and inflammations. The overall positive rate of mSEPT9 for noncolorectal cancers was 19.7% (15/76) (Table 2). Notably, although the overall mSEPT9 positivity in noncolorectal cancers was not as high as CRC, a remarkable positive rate was found in gastric cancer (7/10, 70.0%) (Table 2). Additionally, 13.8% (8/58) of breast cancer patients showed positive mSEPT9, and no positive mSEPT9 was detected in other cancers (0/8), such as pancreatic cancer and melanoma (Table 2). Plasma mSEPT9 was positive in 6.7% (2/30) patients with inflammatory diseases (Table 2), with an AUC of 0.531 (95% CI: 0409–0.653), which indicated mSEPT9 may be not applicable for inflammation detection (Figures 1(c) and 1(d)).

We further evaluated the diagnostic value of circulating mSEPT9 for CRC and beyond CRC using 1/3 algorithm (a high-sensitivity approach). As shown in Supplementary Table S1, mSEPT9 was positive in 80.6% (79/98) CRC cases, and the stage-dependent positivity was 69.6% (16/23), 77.4% (24/31), 93.5 (29/31), and 100% (8/8) with stage I, II, III, and IV, respectively. Additionally, although mSEPT9 positivity in adenoma cases was increased to 16.8% (17/101) with the high-sensitivity algorithm, it was still too low to be used as a biomarker for noninvasive detection of precancerous lesions. The sensitivity, specificity, PPV, and NPV of mSEPT9 for CRC using 1/3 algorithm were 80.61% (95% CI: 71.69%–87.22%), 86.17% (95% CI: 81.37%–89.88%), 69.30% (95% CI: 60.32%–77.02%), and 91.98% (95% CI: 87.82%–94.81%), respectively (Supplementary Table S2).

Among all enrolled subjects, mSEPT9 showed satisfactory diagnostic value for CRC. Its sensitivity, specificity, PPV, and NPV were 61.22% (95% CI: 51.33%–70.27%), 93.7% (95% CI: 91.09%–95.57%), 67.42% (95% CI: 57.13%–76.26%), and 91.9% (95% CI: 89.07%–94.04%) using 2/3 algorithm (Table 3) and 80.61% (95% CI: 71.69%–87.22%), 80.87% (95% CI: 77.03%–84.20%), 47.31% (95% CI: 39.88%–54.85%), and 95.14% (95% CI: 92.54%–96.87%) using 1/3 algorithm (Supplementary Table S2). Taken together, the abovementioned results, plasma mSEPT9 was a remarkable diagnostic biomarker for CRC. Apart from that, mSEPT9 may also be a promising biomarker for gastric cancer, which requires further verification with larger sample sizes.

### 3.2. Correlations between Plasma mSEPT9 Status and Clinicopathologic Characteristics of CRC Subjects

Having determined the diagnostic value of plasma mSEPT9 for CRC, we further explored the correlations between mSEPT9 status and clinicopathologic characteristics. As shown in Table 4, CRC cases with tumor size > 5 cm showed a significantly higher positive rate of mSEPT9 than those with tumor size ≤ 5 cm (77.3% versus 51.7%, *P* = 0.038). Compared with CRC cases with lower histological grade (1 and 2), the subjects with higher histological grade (3 and 4) showed a higher positive rate of mSEPT9 with statistical significance (75.0% versus 51.5%, *P* = 0.046). Interestingly, mSEPT9 positivity in protrude CRC cases was significantly lower than that in ulcerative subjects (33.3% versus 68.9%, *P* = 0.005). No correlation was found between plasma mSEPT9 status and gender, age, tumor location, tumor stage, intravascular tumor thrombus, or tumor nerve invasion of CRC subjects (*P* > 0.05).

### 3.3. Plasma mSEPT9 for Evaluation of Therapeutic Efficacy and Prediction of Impending Recurrence

**Figure 2:**
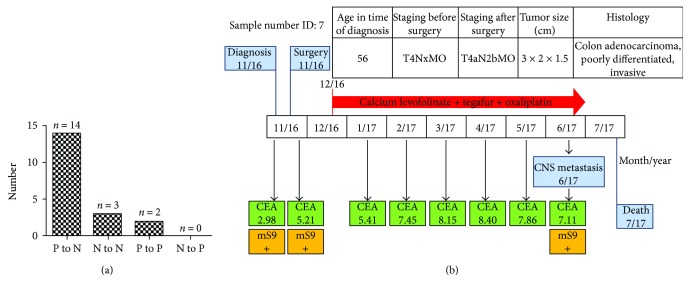
(a) Plasma mSEPT9 status conversion after primary tumor resection in CRC patients. (b) Follow-up of a representative CRC case (sample number ID: 7) with remaining positive mSEPT9 after surgery. (a) P: positive; N: negative. (b) +: positive; −: negative; CNS: central nervous system; CEA: ng/mL, boldface represents positive.

To address the value of plasma mSEPT9 in assessing therapeutic efficacy of CRC, we conducted paired measurement of preoperative and postoperative mSEPT9 in 19 CRC patients underwent curatively intended surgery. Preoperative mSEPT9 was positive in 16 of 19 (84.2%) cases, while CEA was detected elevated only in 6 of 19 cases (31.6%) (*P* = 0.003) (Table 5). In 14 of 16 (87.5%) cases, mSEPT9 converted from preoperatively positive to postoperatively negative (Table 5 and Figure 2(a)). 3 cases with negative preoperative mSEPT9 remained negative after surgery (Table 5 and Figure 2(a)).

It was noteworthy that 2 cases remained positive mSEPT9 despite curatively intended surgery. We speculated that this might accompany with occult tumor cell residue, and postoperative mSEPT9 status may predict impending recurrences or metastases. To address this concern, we followed up these 19 cases for tumor recurrences or metastases for one year. No recurrence was found in the 16 patients with negative postoperative mSEPT9 (follow-up information of one patient was not available) (Table 5). On the contrary, both of the 2 patients with positive postoperative mSEPT9 had recurrences or metastases (Table 5). Based on our data, postoperative mSEPT9 (within 7–14 days) had 100% sensitivity and specificity in predicting impending recurrence (within one year). In comparison, the reliability of postoperative CEA level in CRC recurrences was low, with elevation in 1 of the 2 (50.0%) recurrent CRC patients.

A representative CRC recurrent case (sample number ID: 7) was presented in Figure 2(b). Before surgery, mSEPT9 was positive, but CEA was within the normal reference range. No detectable tumor metastases were found at preoperative and early postoperative stage, with TNM staging of T4NxM0 and T4aN2bM0, respectively. Postoperative mSEPT9 remained positive. About 7 months after surgery, despite the curatively intended surgery and repeated chemotherapy, the patient was found central nervous system (CNS) metastases of CRC, which was further confirmed by CT scan. Considering the postoperative SEPT9 positivity, mSEPT9 status may reflect whether occult tumor cells exist and predict impending tumor recurrence.

### 3.4. Plasma mSEPT9 for Monitoring CRC Recurrence

Having validated the clinical value of mSEPT9 for CRC diagnosis and therapeutic efficacy assessment, we further explored whether it could be utilized as an indicator for recurrence or metastasis. As shown in Table 6, we collected 16 CRC cases who were either recently diagnosed and underwent initial treatment or had been monitored for CRC recurrence. The median period from primary diagnosis and treatment to mSEPT9 measurement was 21 months, ranging from 2 to 102 months. Of the 16 cases, 4 were found recurrences (25.0%) by CT scans, and 3 of the 4 recurrent cases (75.0%) showed positive mSEPT9 around the time of recurrence diagnosis. In comparison, 2 of the 4 recurrent cases (50.0%) showed excessive CEA level. 1 case (sample number ID: 1) with CRC lung metastases showed negative mSEPT9. No evidence of recurrences was found in the remaining 12 cases, which was consistent with the correspondingly negative mSEPT9 status. Overall, our data indicated a good agreement between mSEPT9 status and CRC recurrences (kappa = 0.818, *P* = 0.001).

**Figure 3:**
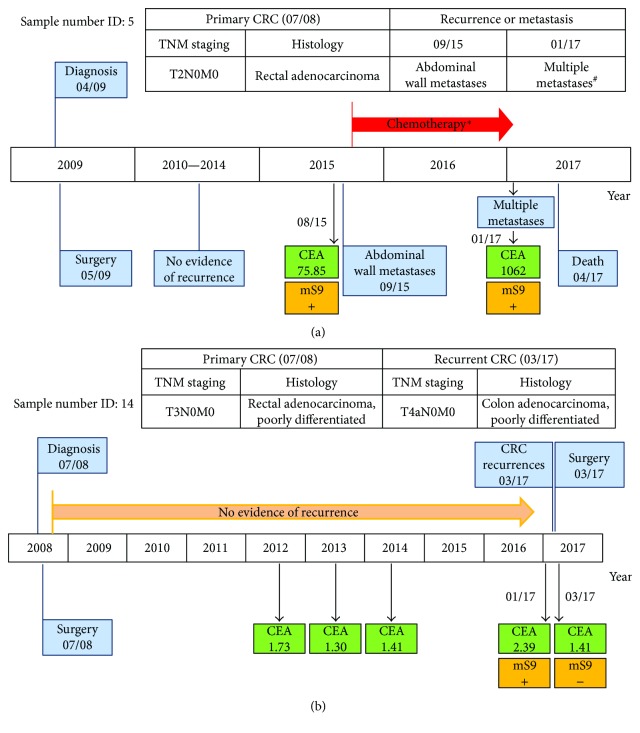
Plasma mSEPT9 for detecting recurrences or metastases in two representative CRC patients during follow-up. (a) Sample number ID: 5. (b) Sample number ID: 14. (a, b) +: positive; −: negative; CEA: ng/mL, boldface represents positive. ^∗^; [Calcium levofolinate + tegafur + oxaliplatin] × 4, [calcium levofolinate + tegafur + irinotecan] × 4, [capecitabine + irinotecan] × 3; ^#^: intrahepatic, peritoneal, retroperitoneal, and subcutaneous metastases.

2 cases with CRC recurrences monitored with mSEPT9 status were presented in Figure 3. In sample number ID: 5, approximately 77 months after primary diagnosis and treatment, both positive mSEPT9 and elevated CEA (75.85 ng/mL) were detected, which agreed with CT scans that indicated abdominal wall metastases (Figure 3(a)). Although the patient underwent multiple chemotherapy from then on, advanced metastases were found 16 months later, including intrahepatic, peritoneal, retroperitoneal, and subcutaneous metastases, accompanied with positive mSEPT9 and excessive CEA (1062 ng/mL) at that time (Figure 3(a)).  Figure 3(b) represents a rectal adenocarcinoma case (sample number ID: 14). 102 months after the primary diagnosis and curatively intended surgery, this patient showed positive mSEPT9 but negative CEA (2.39 ng/mL). About 2 months later, colonoscopy and CT scans indicated recurrent colon adenocarcinoma. Interestingly, mSEPT9 converted to negative after the second surgery, suggesting besides monitoring CRC recurrence, mSEPT9 may also be used for evaluating therapeutic efficacy of CRC recurrences.

## 4. Discussion

Early screening of CRC, accurate assessment of therapeutic efficacy and efficient monitoring of recurrences are urgently needed for improving the treatment outcomes of CRC patients and reducing the disease-related mortality. In this study, we confirmed the value of plasma mSEPT9 for CRC diagnosis, with the sensitivity of 61.22% (95% CI: 51.33%–70.27%) and specificity of 93.7% (95% CI: 91.09%–95.57%) using 2/3 algorithm (Tables 2 and 3; Figures 1(a) and 1(d)). Our statistical analysis also showed that the positive rates of plasma mSEPT9 in CRC patients were correlated with tumor size, histological grade, and general histological type (*P* < 0.05) (Table 4). Remarkably, our data demonstrated that plasma mSEPT9 may also be an effective biomarker in the evaluation of the therapeutic efficacy of curatively intended surgery in CRC patients, as well as in predicting impending recurrences (Table 5 and Figure 2). Moreover, plasma mSEPT9 was also reliable in monitoring CRC recurrences or metastases (Table 6 and Figure 3).

Conventional methods for CRC screening, such as FOBT and colonoscopy, are either not effective enough or invasive [30]. In this research, we evaluated the clinical significance of the noninvasive detection of mSEPT9 in CRC. Our data showed that plasma mSEPT9 had a sensitivity of 61.22% (95% CI: 51.33%–70.27%) for CRC detection using 2/3 algorithm (Table 3), which is higher than other common blood-based biomarker, such as CEA (ranging from 40.9% to 51.8%) [6, 31, 32] and carbohydrate antigen 19-9 (CA19-9) (ranging from 36.4% to 47.8%) [33, 34]. Previous study by Lee et al. [35] showed that fecal immunochemical test (FIT) also exhibited a high sensitivity for CRC, which was similar to plasma mSEPT9. However, clinical compliance of this stool-based test was low because of its inconvenience and requirement of repeated tests [27]. The sensitivity of plasma mSEPT9 for CRC using 2/3 algorithm in our study is consistent with the data in a quantitative meta-analysis by Zhang et al. [36], which showed that plasma mSEPT9 sustained a pooled sensitivity of 64% (95% CI: 59%–68%) for CRC detection in the Asian-based population. Previous studies showed that the 1/3 algorithm draws higher sensitivity and lower specificity than the 2/3 algorithm in mSEPT9 test, with the sensitivity ranging from 68% (95% CI: 53% -80%) to 95.6% (95% CI: 89.2%–98.8%) for CRC detection [6, 37–39]. Consistent with these data, when using 1/3 algorithm in our analysis, the sensitivity reached 80.61% (95% CI: 71.69%–87.22%) and the specificity was 86.17% (95% CI: 81.37%–89.88%) (Supplementary Table S2). More importantly, when using 1/3 algorithm, 69.6% (16/23) and 77.4% (24/31) specimens were mSEPT9 positive for stage I and II CRC, respectively (Supplementary Table S1), suggesting that plasma mSEPT9 measurement using 1/3 algorithm may also be an early-stage CRC screening approach. These data indicate that different algorithms may be applicable to different test purposes: 1/3 algorithm is suitable for early cancer screening, while 2/3 algorithm is superior in disease detection. Taken together, plasma mSEPT9 is a sensitive and specific biomarker for CRC, and this blood-based test is patient friendly, noninvasive, and with high compliance.

Beyond CRC, our data indicated that mSEPT9 may also be utilized as a potential biomarker for gastric cancer, with a positive rate of 70% (7/10) (Table 2). As CRC and gastric cancer are both gastroenteric tumor, they share some molecular characteristics including microsatellite instability, hypermethylation, and gene mutations [40], which may explain the similarly high mSEPT9 positivity. Of note, 8 out of 10 enrolled gastric cancer subjects were gastric cardia cancer. This limited case number may not be sufficient to determine whether this high detection rate is associated with gastric cancer or specifically for gastric cardia cancer. Further studies with larger sample sizes will be precious to verify the diagnostic value of mSEPT9 for gastric cancer, especially for gastric cardia cancer. Consistent with previous reports [41, 42], our data showed a low positive rate (7.9% using 2/3 algorithm and 16.8% using 1/3 algorithm) of plasma mSEPT9 for adenoma, suggesting the inapplicability of mSEPT9 for premalignant adenoma detection.

Few studies have been conducted to investigate the association between mSEPT9 status and clinicopathologic characteristics of CRC patients. In our research, we found mSEPT9 positivity was higher in the CRC subjects with larger tumor size (>5 cm) than those with smaller tumor size (≤5 cm) (Table 4). It may be because less circulating cell-free DNA was released from smaller tumor below the detection limit of mSEPT9 with the current method. Unprecedentedly, our data revealed a high association between mSEPT9 status and histological grade. The positive detection rate of mSEPT9 was significantly higher in subjects with higher histological grade (3 and 4) than those with lower histological grade (1 and 2) (Table 4). This result suggests that mSEPT9 positivity may represent high malignancy and possibly be associated with poor prognosis.

The histopathologic characteristics of CRC patients associated with higher recurrent risk only indicates a propensity for metastasis but were not able to address whether metastatic tumor cells have been seeded at the time of surgery [43]. Our results revealed that the persistent positivity of plasma mSEPT9 after curatively intended surgery was highly correlated with impending recurrences or metastases within one year, whereas mSEPT9 positive to negative conversion indicated free from recurrences (Table 5 and Figure 2). From this perspective, mSEPT9 may serve as a reliable biomarker for assessing therapeutic efficacy for CRC patients whose preoperative mSEPT9 was positive. These findings also suggest that postoperative mSEPT9 status may be a direct indication whether occult tumor cells remain in the patient and predict impending recurrent diseases after surgery. It is also meaningful that mSEPT9 conversion was detected within only 7–14 days. For clinical management, this is a relevant time window for assessing the recurrent risk of CRC and deciding whether adjuvant treatment would be necessary after curative intended surgery.

The timely detection of CRC recurrences or metastases in postoperative patients is of particular importance. Our data showed a good agreement between the mSEPT9 status and CRC recurrences (kappa = 0.818, *P* = 0.001) (Table 6 and Figure 3), suggesting that the mSEPT9 test may efficiently identify CRC recurrence. It has to be noted that 1 case with lung metastases (sample number ID: 1) had negative mSEPT9. Unfortunately, we were not able to obtain the preoperative mSEPT9 status of this case. Its need to clarify whether mSEPT9 could be used for detection of recurrent diseases in CRC cases whose preoperative mSEPT9 status is negative. Another limitation is that we mostly detected the mSEPT9 status around the time when the recurrences or metastases were diagnosed. Its also need to further elucidate whether mSEPT9 test has the potential to provide clinically relevant lead times compared with the conventional diagnostic modalities for the incipient recurrence detection.

In summary, our results showed that plasma mSEPT9 was a promising biomarker in CRC diagnosis. Notably, we revealed significant association between mSEPT9 status and tumor size, histological grade, and general histological type. Plasma mSEPT9 was also valuable for evaluating the therapeutic efficacy of curatively intended surgery and predicting impending recurrences or metastases. Postoperative mSEPT9 during follow-up served as a significant indicator for CRC recurrences. The limitations of this study include qualitative rather than quantitative detection of mSEPT9 and limited number of patients enrolled in a single hospital. Further studies are required to confirm the promising findings.

## Figures and Tables

**Table 1:** Demographic and clinicopathologic characteristics of enrolled subjects for evaluating diagnostic value of plasma mSEPT9.

Characteristics	*N*	Gender		Age				Location	
		Male	Female	<50	50–59	60–69	≥70	Colon	Rectum
CRC	98	61	37	14	22	29	33	18	80
Stage 0	3	2	1	1	1	0	1	1	2
Stage I	23	16	7	4	7	4	8	7	16
Stage II	31	20	11	5	4	10	12	4	27
Stage III	31	19	12	2	9	11	9	5	26
Stage IV	8	3	5	2	0	3	3	1	7
Unknown	2	1	1	0	1	1	0	0	2
Adenomas	101	71	30	30	21	34	16	84	17
Noncolorectal cancer	76	15	61	33	18	17	8	NA	NA
Gastric cancer	10	8	2	0	0	4	6	NA	NA
Breast cancer	58	0	58	31	15	12	0	NA	NA
Others	8	7	1	2	3	1	2	NA	NA
Inflammation	30	14	16	8	7	13	2	NA	NA
Gastroenteritis	26	13	13	8	5	14	2	NA	NA
Others	4	1	3	0	2	2	0	NA	NA
NED	253	139	114	32	144	54	23	NA	NA
Total	558	300	258	117	212	147	82	NA	NA

CRC: colorectal cancer; NED: no evidence of diseases; NA: not applicable.

**Table 2:** Positive rate of plasma mSEPT9 in each enrolled group using 2/3 algorithm.

Characteristics	*N*	mSEPT9		*P* value^†^	*P* value^‡^
		*N*	%		
CRC	98	60	61.2	<0.001	Ref.
Adenomas	101	8	7.9	0.006	<0.001
Noncolorectal cancer	76	15	19.7	<0.001	<0.001
Gastric cancer	10	7	70.0		
Breast cancer	58	8	13.8		
Other cancer	8	0	0.0		
Inflammation	30	2	6.7	0.125	<0.001
Gastroenteritis	26	2	7.7		
Other Inflammation	4	0	0.0		
NED	253	4	1.6	Ref.	<0.001

CRC: colorectal cancer; NED: no evidence of diseases; ^†^: compared with NED (ref.); ^‡^: compared with CRC (ref.).

**Table 3:** Diagnostic test evaluation of plasma mSEPT9 for CRC using 2/3 algorithm.

	mSEPT9^†^	mSEPT9^‡^
Sensitivity (95% CI)	61.22% (51.33%–70.27%)	61.22% (51.33%–70.27%)
Specificity (95% CI)	98.42% (96.01%–99.38%)	93.70% (91.09%–95.57%)
PPV (95% CI)	93.75% (85.00%–97.54%)	67.42% (57.13%–76.26%)
NPV (95% CI)	86.76% (82.35%–90.20%)	91.90% (89.07%–94.04%)

PPV: positive predictive value; NPV: negative predictive value; CI: confidence interval; ^†^: mSEPT9 in subjects with colorectal cancer versus no evidence of diseases; ^‡^: mSEPT9 in subjects with colorectal cancer versus all the other enrolled subjects including adenoma, noncolorectal cancer, inflammation, and no evidence of disease samples.

**Table 4:** Correlations between plasma mSEPT9 and clinicopathologic characteristics of CRC patients.

Characteristics	Case	mSEPT9 positive case		*P* value
	*N*	*N*	%	
All cases	98	60	61.2	
Gender				
Male	61	33	54.1	0.063
Female	37	27	73.0	
Age (years)				
≤60	38	21	55.3	0.335
>60	60	39	65.0	
Tumor size (cm)				
≤5	58	30	51.7	0.038
	22	17	77.3	
Unknown	18	13	72.2	
Location				
Colon	18	14	77.8	0.111
Rectum	80	46	57.5	
General histological type				
Ulcerative	45	31	68.9	0.005^†^
Protrude	24	8	33.3	
Others	3	1	33.3	
Unknown	26	20	76.9	
Histological grade				
1 + 2	66	34	51.5	0.046
3 + 4	24	18	75.0	
Unknown	8	8	100.0	
Stage				
0	3	1	33.3	0.376
I	23	11	47.8	
II	31	19	61.3	
III	31	20	64.5	
IV	8	7	87.5	
Intravascular tumor thrombus				
Negative	25	18	72.0	0.055
Positive	57	28	49.1	
Unknown	16	14	87.5	
Nerve invasion				
Negative	34	23	67.6	0.187
Positive	22	11	50.0	
Unknown	16	11	68.8	

^†^: mSEPT9 in ulcerative CRC versus protrude CRC.

**Table 5:** Therapeutic efficacy follow-up after curatively intended surgery by mSEPT9 assay.

Number ID	Gender	Age	TNM staging	mSEPT9		CEA		Recurrent status^†^
				Pre	Post	Pre	Post	
1	Male	74	T3N1bM0	+	−	**8.64**	1.24	NER
2	Female	68	T3N1aM0	+	−	**11.23**	3.32	NER
3	Male	72	T2N1aM0	+	+	2.04	1.11	Liver metastases
4	Female	63	T3N0M0	+	−	0.61	0.76	NER
5	Male	54	T2N1aM0	+	−	4.29	1.62	NER
6	Male	56	T3N1bM0	+	−	2.12	1.96	NER
7	Male	56	T4aN2bM0	+	+	2.98	**5.21**	CNS metastases
8	Female	57	T3N1aM0	+	−	1.88	0.68	NER
9	Female	61	T4aN1aM0	+	−	1.22	1.24	NER
10	Male	53	T4aN1aM0	−	−	1.71	1.42	NER
11	Male	55	T3N0M0	−	−	**7.75**	2.01	NER
12	Male	63	T3N0M0	+	−	2.32	1.25	NER
13	Male	53	T1N0M0	+	−	4.25	1.89	NER
14	Female	66	T3N0M0	+	−	**26.69**	1.84	NER
15	Male	77	T3N0M0	+	−	4.12	4.32	NER
16	Female	78	T3N0M0	+	−	2.16	0.82	Unknown
17	Male	82	T3N0M0	+	−	**11.08**	4.47	NER
18	Male	40	T2N0M0	−	−	1.93	2.12	NER
19	Female	44	T3N0M0	+	−	**7.89**	0.98	NER

Pre: preoperative; Post: postoperative; NER: no evidence of recurrence; CNS: central nervous system; +: positive; −: negative; boldface in CEA column represents positive; ^†^: CRC recurrences or metastases during one-year postoperative follow-up.

**Table 6:** CRC recurrence detection by plasma mSEPT9 during follow-up.

Number ID	Gender	Age	TNM staging	Treat.	Period^†^ (months)	mSEPT 9	CEA	Recurrent status
1	Female	61	T4aN1bM0	S + C	22	−	2.53	Lung metastases
2	Female	42	T2N0M0	S	16	−	1.58	NER
3	Male	59	T3N0M0	S	9	−	2.04	NER
4	Male	53	T2N0M0	S	2	−	2.40	NER
5	Female	64	T2N0M0	S	77	+	**75.85**	Abdominal wall metastases
6	Female	63	T3N1aM0	S + C	36	−	1.68	NER
7	Female	52	T3N1aM0	S + C	8	−	2.32	NER
8	Male	58	T4aN0M0	S + C	24	−	2.62	NER
9	Male	56	T3N0M0	S	48	−	2.25	NER
10	Male	58	T2N0M0	S + C	8	−	1.59	NER
11	Female	62	T3N0M0	S	24	−	4.29	NER
12	Female	61	TisN0M0	S	34	−	1.70	NER
13	Male	42	T3N0M0	S + C	20	−	0.76	NER
14	Male	60	T3N0M0	S	102	+	1.41	Recurrent CRC
15	Male	43	T2N0M0	S	3	−	2.59	NER
16	Female	60	T4aN0M0	S + C	11	+	**7.59**	Liver metastases

Treat.: treatment; S: curatively intended surgery; C: chemotherapy; NER: no evidence of recurrence; +: positive; −: negative; boldface in CEA column represents positive; ^†^: period after treatment.

## Supplementary Materials

Supplementary Materials Table S1: positive rate of plasma mSEPT9 in each enrolled group using 1/3 algorithm. Table S2: diagnostic test evaluation of plasma mSEPT9 for CRC using 1/3 algorithm.
