# Supplementary material for: Cell-Free Circulating Methylated SEPT9 for Noninvasive Diagnosis and Monitoring of Colorectal Cancer
Source: Dis Markers. 2018 Apr 23;2018:6437104. doi: 10.1155/2018/6437104 (PMC5937566; doi:10.1155/2018/6437104)
Supplement: Supplementary Materials — Table S1: positive rate of plasma mSEPT9 in each enrolled group using 1/3 algorithm. Table S2: diagnostic test evaluation of plasma mSEPT9 for CRC using 1/3 algorithm. [file 6437104.f1.pdf]

Supplementary Files

Supplementary Table S1 Positive rate of plasma mSEPT9 in each enrolled group using 1/3 algorithm.

Characteristics	N	mSEPT9	
		N	%
CRC	98	79	80.6
0	3	1	33.3
Ⅰ	23	16	69.6
Ⅱ	31	24	77.4
Ⅲ	31	29	93.5
Ⅳ	8	8	100.0
Unknown	2	1	50.0
Adenomas	101	17	16.8
Non-colorectal Cancer	76	29	38.2
Gastric cancer	10	8	80.0
Breast cancer	58	20	34.5
Other cancer	8	1	12.5
Inflammation	30	7	23.3
Gastroenteritis	26	6	23.1
Other Inflammation	4	1	25.0
NED	253	35	13.8

CRC, colorectal cancer; NED, no evidence of diseases; †, compared with NED (Ref.); ‡, compared with CRC (Ref.).

Supplementary Table S2 Diagnostic test evaluation of plasma mSEPT9 for CRC using 1/3 algorithm.

	mSEPT9†	mSEPT9‡
Sensitivity (95% CI)	80.61% (71.69% - 87.22%)	80.61% (71.69% - 87.22%)
Specificity (95% CI)	86.17% (81.37% - 89.88%)	80.87% (77.03% - 84.20%)
PPV (95% CI)	69.30% (60.32% - 77.02%)	47.31% (39.88% - 54.85%)
NPV (95% CI)	91.98% (87.82% - 94.81%)	95.14% (92.54% - 96.87%)

PPV, positive predictive value; NPV, negative predictive value; CI, confidence interval; †, mSEPT9 in subjects with colorectal cancer versus no evidence of diseases; ‡, mSEPT9 in subjects with colorectal cancer versus all the other enrolled subjects including adenoma, non-colorectal cancer, inflammation, and no evidence of disease samples.
